# Shortened chordae tendineae of the tricuspid valve with right ventricular dysfunction caused by acute myocarditis lead to cardiogenic shock during pericardial drainage: a case report

**DOI:** 10.1093/omcr/omac097

**Published:** 2022-09-26

**Authors:** Motoaki Higuchi, Kisato Mitomi, Yoshiro Chiba

**Affiliations:** Department of Cardiology, Mito Saiseikai General Hospital, Mito, Ibaraki Prefecture, Japan; Department of Cardiovascular Surgery, University of Tsukuba Hospital, Tsukuba, Ibaraki Prefecture, Japan; Department of Cardiology, Mito Saiseikai General Hospital, Mito, Ibaraki Prefecture, Japan

## Abstract

A 63-year-old woman was admitted to the hospital with general malaise and pericardial and thoracoabdominal effusions of unknown cause. After pericardial drainage for drug-resistant right heart failure, she developed right ventricular (RV) dysfunction and cardiogenic shock caused by severe tricuspid regurgitation (TR). Findings during emergency surgery included tricuspid valve (TV) junction failure caused by shortening of the chordae tendineae of the TV, which is an organic abnormality. Additionally, myocardial biopsy results revealed myocarditis. Although acute myocarditis developed with RV dysfunction, pericardial effusion suppressed venous return, which temporarily improved her pathological condition. However, RV dysfunction and severe TR were thought to have manifested after the venous return suppression was alleviated by pericardial drainage. Because venous return changes significantly after pericardial drainage, it is necessary to examine the need for drainage and re-evaluate the post-operative RV system.

## INTRODUCTION

Pericardial effusion suppresses venous return, which may conceal right ventricular (RV) or tricuspid valve (TV) dysfunction. If pericardial drainage is performed for such cases, then increased venous return may reveal RV dysfunction and tricuspid regurgitation (TR), and the risk of cardiogenic shock may develop. We present the case of a patient with shortened chordae tendineae of the TV in whom pericardial effusion concealed severe TR. Drainage revealed TR, which was accompanied by RV dysfunction secondary to acute myocarditis and cardiogenic shock.

## CASE REPORT

**Figure 1:**
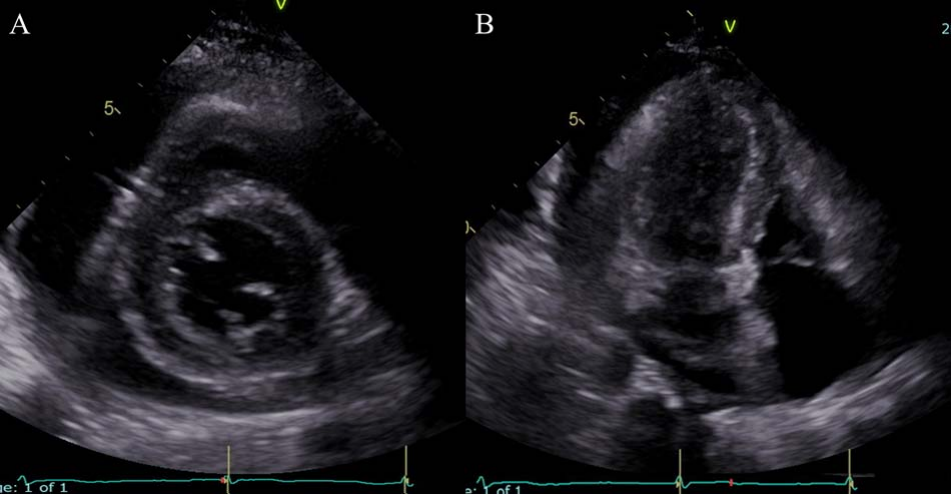
TTE at the time of admission; (**A**) Parasternal short-axis view: pericardial effusions of 7 mm around the right ventricle and 18 mm around the LV; (**B**) modified apical four-chamber view; TR insufficiency is unclear.

A 63-year-old woman with no apparent history of rheumatic fever and cardiac diseases presented with general malaise and was hospitalized. On admission, her blood pressure and pulse were 120/87 mmHg and 60 beats/minute, respectively. The blood test revealed the following results: brain natriuretic peptide, 186 pg/ml (reference, 0–18.4 pg/ml); cardiac troponin I, 0.038 ng/ml (reference, 0–0.025 ng/ml); creatinine kinase (CPK), 228 U/l (reference, 29–248 U/l); CPK-MB, 30 U/l (reference, 5–25 U/l) and C-reactive protein, 2.69 mg/dl (reference, 0–0.14 mg/dl). Furthermore, an electrocardiogram (ECG) revealed a marked PQ duration of 0.48 msec; however, no ST-segment elevation was noted by an 18-lead ECG. Transthoracic echocardiography (TTE) revealed normal cardiac function and pericardial effusions of 7 mm around the RV and 18 mm around the left ventricle (LV; Fig. 1).

**Figure 2:**
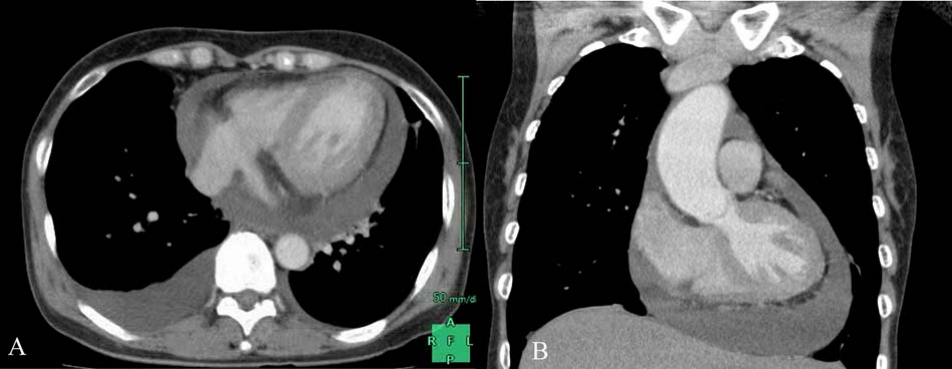
Thoracic and abdominal CT performed at admission; (**A**) moderate pericardial effusion and right pleural effusion at the transverse section; (**B**) moderate pericardial effusion at the coronal section.

**Figure 3:**
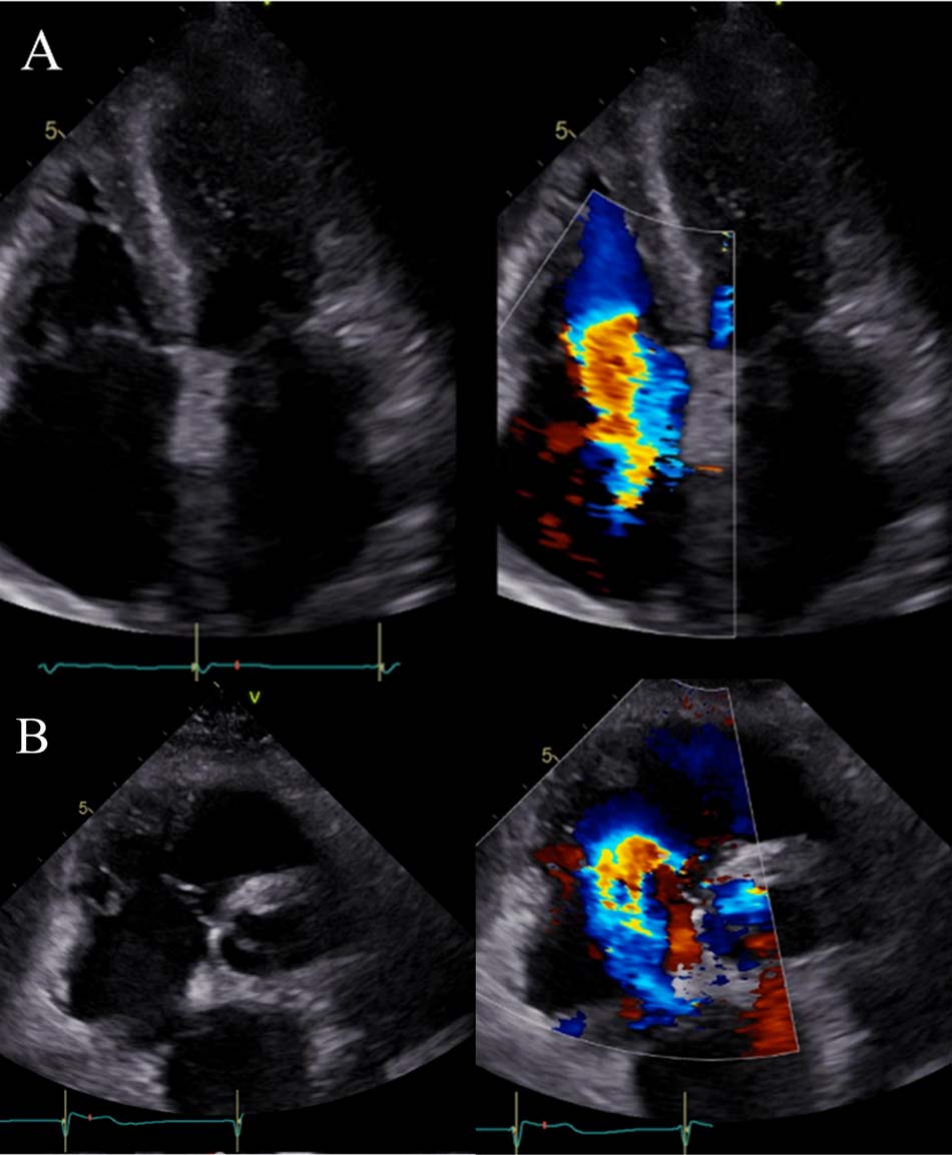
TTE after pericardial drainage; (**A**) apical four-chamber view: severe TR signal using Doppler echo with TV insufficiency during systole; (**B**) parasternal short-axis view at the aortic valve level: TV insufficiency during systole; a severe TR signal is noted secondary to TV insufficiency using Doppler echo.

**Figure 4:**
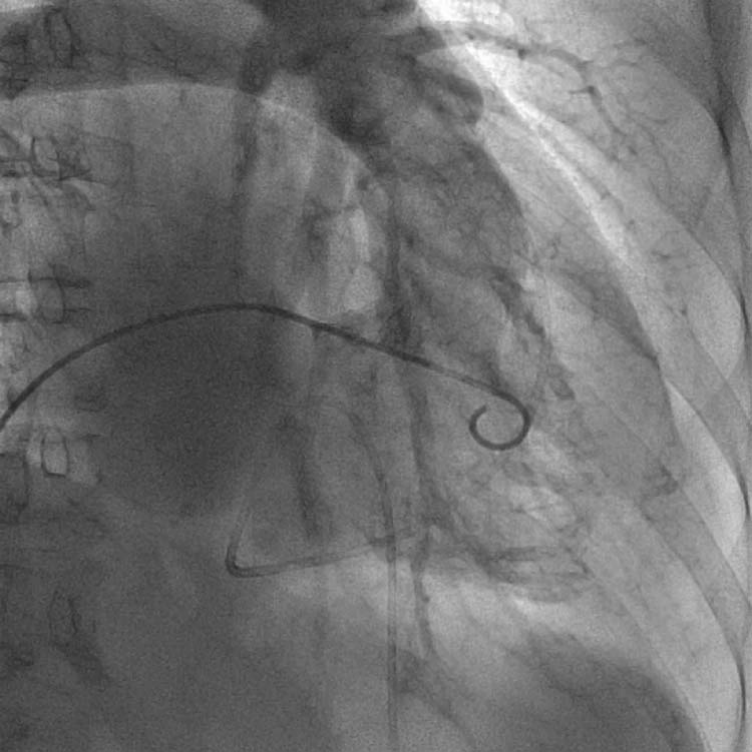
Right ventriculography image obtained in the 30° right anterior oblique view shows a 5-Fr pigtail catheter inserted via the right femoral vein approach; the contrast concentrations in the right ventricle and right atrium appear nearly identical.

**Figure 5:**
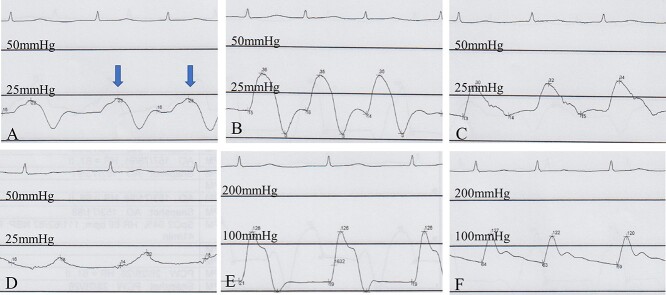
Intracardiac pressure waveform with a Swan–Ganz catheter; (**A**) the right atrial mean pressure is 15 mmHg; arrows indicate an increase in the v-wave height; (**B**) the RV systolic pressure is 34 mmHg, whereas the RV end-diastolic pressure is 15 mmHg; (**C**) the pulmonary artery pressure is 34/15 mmHg; (**D**) the pulmonary artery wedge pressure is 18 mmHg; (**E**) the LV systolic pressure is 123 mmHg, whereas the LV end-diastolic pressure is 20 mmHg; (**F**) the aortic pressure is 119/60 mmHg.

**Figure 6:**
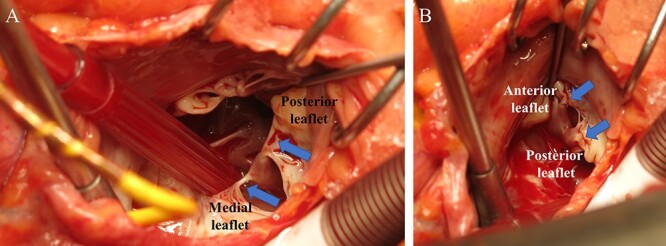
Surgical findings during TVR; (**A**) arrows indicate the shortened TV chordae tendineae; (**B**) the arrow indicates the shortened TV chordae tendineae; the chordae tendineae were shortened to the extent that the papillary muscle and leaflet were almost attached.

**Figure 7:**
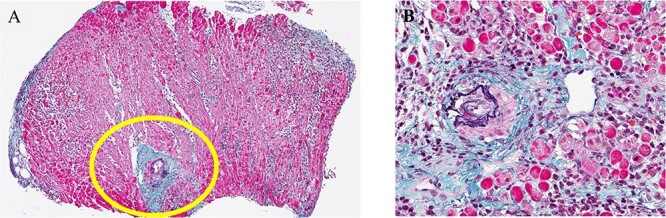
Histopathological findings after RV myocardial biopsy; (**A**) infiltration of T lymphocytes and disappearance of cardiomyocytes are observed in some parts of the myocardial tissue; (**B**) enlarged image of the circled region in (A); vasculitis and fibrosis of the cardiomyocytes are observed.

There was no obvious evidence of valvular diseases, including TR. Furthermore, computed tomography (CT) revealed pericardial and thoracoabdominal effusions (Fig. 2). On the day of admission, her hemodynamic status was stable and treatment was initiated with diuretics. However, the next day, her symptoms worsened and urine output was poor; therefore, pericardial drainage was performed to remove 320 ml of yellow and turbid pericardial fluid. The subjective symptoms improved thereafter; however, she experienced cardiogenic shock the day after treatment. TTE revealed that the TV ring diameter had enlarged from 33 mm (at admission) to 45 mm and that severe TR had developed (Fig. 3). Emergency catheterization was performed to evaluate hemodynamics, coronary artery lesions and valvular diseases. Swan–Ganz catheterization revealed cardiac output, cardiac index and SvO_2_ of 2.35 l/min, 1.68 l/min/m^2^ and 45%, respectively. Increased right atrial pressure and an increased v-wave were also observed (Fig. 4). Right ventriculography (Fig. 5) revealed a 30% decrease in the RV ejection fraction and severe TR (Seller’s classification grade 3). The coronary artery and LV ejection fraction were normal, and mild mitral regurgitation was noted. Furthermore, a myocardial biopsy of the RV septum was performed. Despite insertion of an intra-aortic balloon pump, her hemodynamic status did not improve. Because of circulatory failure caused by severe TR, TV replacement (TVR) surgery was performed (27 mm; MAGNA MITRAL EASE Edwards Lifesciences, Irvine, CA, USA). Intraoperatively, the chordae tendineae of the TV were markedly shortened (Fig. 6). Thereafter, a biopsy revealed T-lymphocyte infiltration and myocardial fibrosis (Fig. 7). The patient was diagnosed with acute myocarditis. She was discharged 45 days after admission and remained free of cardiovascular events for the next 3 years.

## DISCUSSION

This case comprised underlying organic abnormalities of the chordae tendineae of the TV and incidental presentation with RV dysfunction secondary to acute myocarditis. Pericardial drainage, which was performed as treatment, caused the TR and RV dysfunction to manifest and led to cardiogenic shock. Pericardial drainage reportedly causes preload variation before and after drainage in the right heart system, which is susceptible to pericardial cavity pressure; furthermore, the RV diastolic volume increases after pericardial drainage [1].

Compared to in the general population, the right heart system was more susceptible to venous return of this patient with shortened TV chordae tendineae, which led to the enlargement of the right heart system and severe TR caused by TV junction insufficiency. Acute myocarditis can be fatal, mainly because of pump failure and fatal arrhythmias secondary to LV dysfunction [2]; however, RV dysfunction secondary to myocarditis alone can be serious in patients with organic abnormalities (such as TV tendon shortening). It should be noted that acute myocarditis causing only RV dysfunction may also cause hemodynamic instability.

Severe RV function and TV dysfunction have occurred after pericardial drainage [3]. Our patient was at high risk because her symptoms worsened after admission and pericardial drainage was a priority. If the presence and severity of myocarditis could have been ascertained by gadolinium contrast-enhanced magnetic resonance imaging (MRI; because of the possibility of acute myocarditis based on the presence of increased myocardial deviation enzymes and atrioventricular conduction delay on admission), then pericardial drainage might have been avoided. Colchicine administration is effective for pericardial effusion caused by pericarditis [4]. In this case, acute myocarditis was not diagnosed initially; TVR was performed to manage TV chordae tendineae shortening, which is an organic disorder. However, if the transient RV dysfunction secondary to acute myocarditis had been an exacerbating factor, then medical therapy or extracorporeal membrane oxygenation would have improved the situation, and TVR might not have been necessary. For a previously reported case, medical therapy achieved an improvement in cardiogenic shock caused by RV dysfunction combined with TV dysfunction after pericardial drainage [5]. Conversely, TVR still may have been necessary for the present case because of the tendon abnormality.

TR is classified as primary and secondary. Primary TR, a structural abnormality of the TV, accounts for ~8% of all TR cases [6]; possible causes include Epstein’s malformation, infective endocarditis, pacemaker leads, chest trauma and rheumatic fever. Conversely, congenital TR with structural abnormalities but without a history of these conditions has also been reported; some cases have been diagnosed at an advanced age [7, 8]. Our patient likely had congenital TR.

Pericardial drainage is necessary for the treatment and identification of the cause. If the TV involves an organic abnormality (as in this case), or if acute myocarditis has spread to the RV, then venous return will have a strong, negative hemodynamic effect. Screening with MRI and TTE may help reduce the risks of TR and RV dysfunction after pericardial drainage.

## CONFLICT OF INTEREST STATEMENT

None declared.

## FUNDING

None.

## ETHICAL APPROVAL

Institutional review board approval was not required for this case report.

## CONSENT

Written informed consent for the publication of this case was obtained from the patient.

## GUARANTOR

Higuchi Motoaki.
